# Congenital hepatic haemangioma leading to multiple organ failure in a neonate

**DOI:** 10.1259/bjrcr.20150399

**Published:** 2016-05-11

**Authors:** Sarah Aouni, Vinciane Vlieghe, Valérie Segers, Catherine Christophe

**Affiliations:** ^1^ Pediatric Radiology Department, Hôpital Universitaire des Enfants Reine Fabiola, Université Libre de Bruxelles, Brussels, Belgium; ^2^ Neonatalogy Department, Hôpital Universitaire des Enfants Reine Fabiola, Université Libre de Bruxelles, Brussels, Belgium; ^3^ Pathology Department, Hôpital Universitaire des Enfants Reine Fabiola, Université Libre de Bruxelles, Brussels, Belgium

## Abstract

We report a case of a premature male newborn who died from multiple organ failure due to a large congenital hepatic haemangioma that was diagnosed by imaging. Congenital haemangioma is a vascular tumour. The liver is the second organ involved after the skin. This tumour can be asymptomatic but can also lead to death.

## Clinical presentation

A premature male was admitted to our hospital following detection of a hepatic mass on ultrasound associated with severe liver failure, haemolysis and thrombopenia on blood tests.

He was delivered by caesarean section at 35 weeks gestational age because of abnormal cardiac rhythm. The pregnancy was marked by polyhydramnios. At birth, the baby was in asystolia, with an Apgar score of 0/4/5.

Despite spontaneous breathing, a tracheal intubation was necessary because of a huge hepatomegaly compressing the lungs. Clinical examination also revealed hydrops and systemic hypotension. Relevant abnormalities found on echocardiography were ventricular hypertrophy and pulmonary arterial hypertension.

## Imaging findings

**Figure 1.**
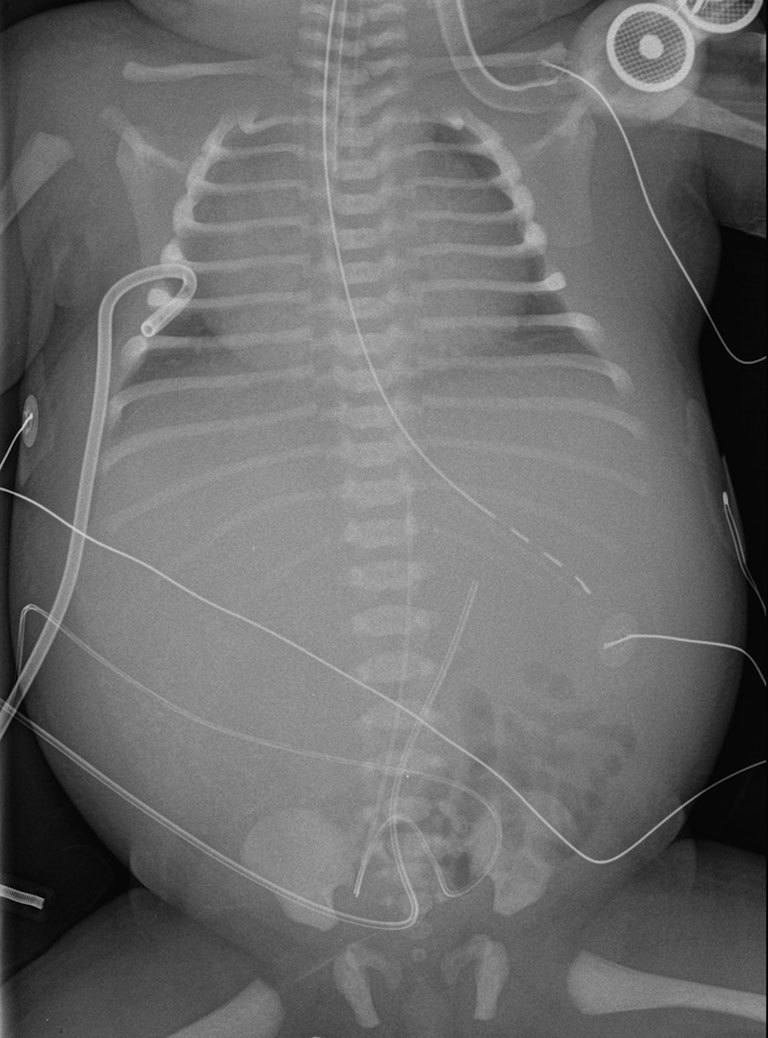
X-ray of the chest and abdomen showed cardiomegaly, acute pulmonary and soft tissues oedema, and an opacity related to hepatomegaly.

X-rays on admission showed signs of cardiac failure and hepatomegaly (Figure 1).

**Figure 2.**
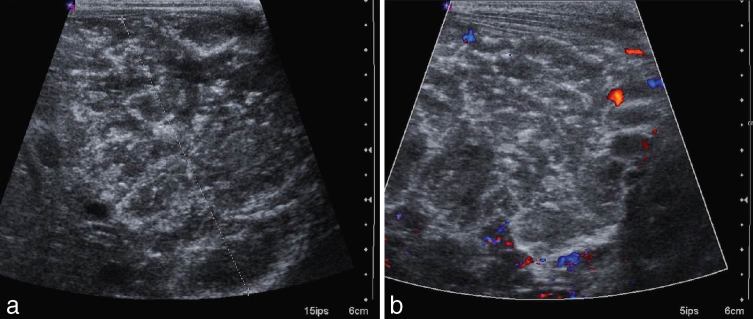
Ultrasound demonstrated a hyperechoic, heterogeneous hepatic mass (between calipers) (a) with moderate vascularization on Doppler ultrasound (b) and small calcifications.

The ultrasound (Figure 2) was repeated and showed a large hyperechoic and heterogeneous hepatic mass, measuring 89 mm in the cephalocaudal axis, 70 mm in the anterior–posterior axis and 60 mm wide. The mass contained small calcifications and moderate vascularization. Both hepatic veins and artery were enlarged, especially the right hepatic vein. The portal vein was of normal diameter.

**Figure 3.**
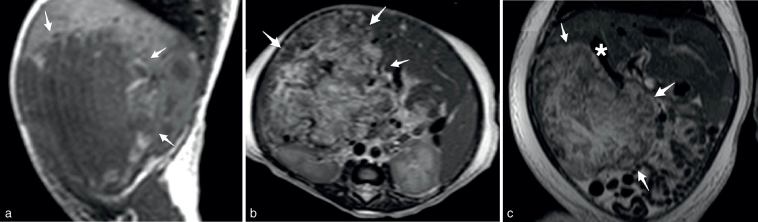
MRI showed a heterogeneous hepatic mass (arrows) in the right lobe, mostly hypointense on *T*_1_ (a, parasagittal view) and hyperintense on *T*_2_ weighted (b, axial view; c, frontal view) images, with enlarged hepatic veins (asterisk).

An MRI (Figure 3) was performed without contrast, because of renal failure. The hepatic mass was irregular and occupied segments V, VI and VII. The mass was hypointense on *T*_1_ weighted imaging, heterogeneous and hyperintense on *T*_2_ weighted imaging and contained small intramural vessels. Hepatic parenchyma was otherwise normal.

**Figure 4.**
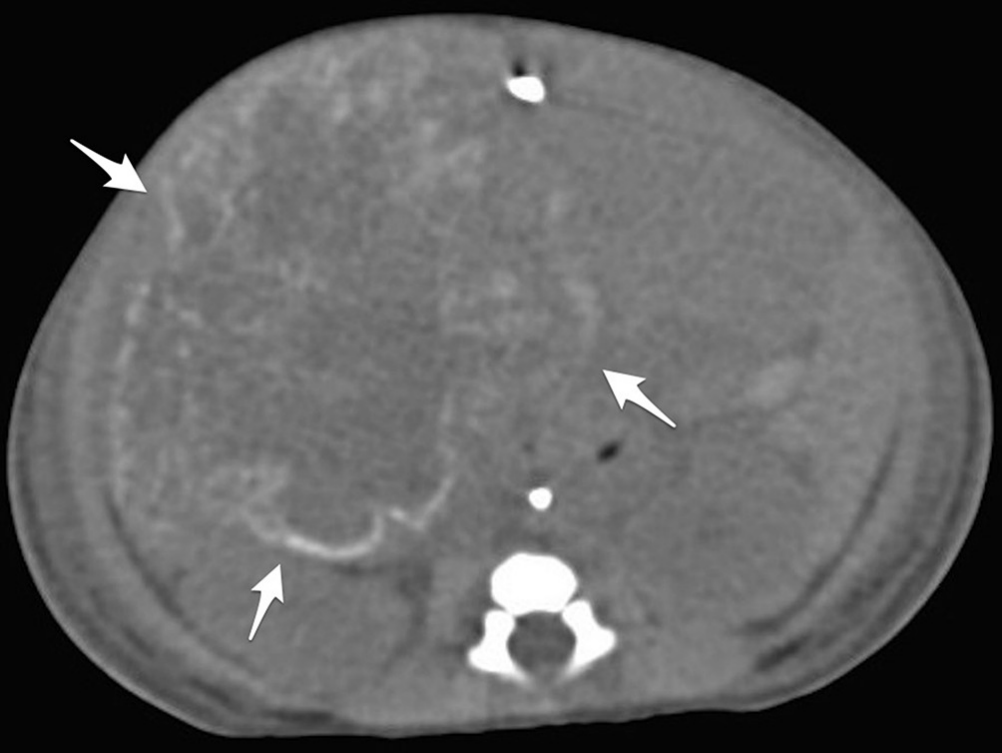
Non-enhanced CT scan showed predominantly peripheral calcifications (arrows).

Neither arteriovenous nor portovenous shunt was detected by ultrasound or MRI. A non-enhanced CT scan (Figure 4) confirmed predominantly peripheral fine calcifications, sometimes “eggshell-like” (CT dose index 2.66 mGy). No pulmonary metastasis was found.

## Differential diagnosis

Suspected diagnosis was congenital haemangioma (CH) or hepatoblastoma.

## Investigations

Tumour-associated markers (α-fetoprotein, neuron-specific enolase, β-human chorionic gonadotropin and urinary catecholamines) were normal.

## Outcome

**Figure 5.**
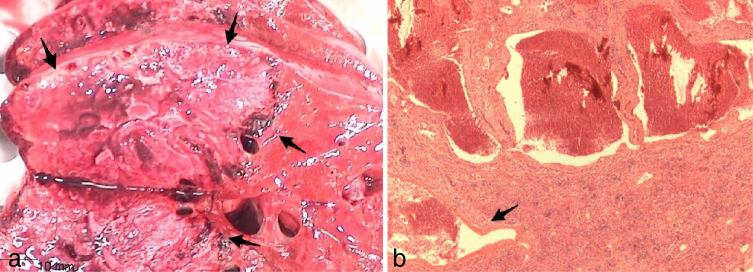
(a) Gross anatomy, large necrotic hepatic mass (arrows) of the right lobe, adjacent to normal parenchyma. (b) Histological specimen, large intratumoral necrosis with peripheral varying-sized vascular lakes (arrow). The glucose transporter-1 marker was negative.

The baby developed disseminated intravascular coagulopathy, haemodynamic instability because of liver failure, and haematologic disorders and acute pulmonary oedema owing to prerenal acute kidney injury with anuria. He finally died on day 3 following pulmonary oedema secondary to multiple organ failure. Fluid overload was suspected to be due to undetected intratumoral arteriovenous shunts. Histology (Figure 5) found a giant congenital hepatic haemangioma (CHH).

## Discussion

### Epidemiology

A haemangioma is a benign vascular tumour occurring in foetuses and infants before the age of 12 months.^1,2^ A haemangioma involves mostly the skin, but the liver is the second involved organ.^2^ The most common neonatal hepatic tumour is a haemangioma.^2^ The most common subtype of haemangioma is infantile haemangioma (IH). IH is rarely present at birth unlike CH, which is typically present at birth.^1,3^ As CH begins to grow *in utero,* it can be detected during foetal ultrasound.^1,2,4^ Histology shows a few differences between IH and CH. Especially, IH is glucose transporter-1 (GLUT1) positive, whereas CH is GLUT1 negative.^2^

CHs can be divided into three subtypes: rapidly involuting congenital haemangioma (RICH), non-involuting congenital haemangioma (NICH) and the recently described partially involuting congenital haemangioma (PICH).^1^ Neither subtype grows after birth and some CH stop growing during the third trimester.^3^ RICH regresses before the age of 14 months, whereas NICH does not regress, but grows proportionally with the child.^3–5^ None of them show any gender predominance.^4,6^ CHH is usually solitary and is generally classified as a RICH.^2–4,6^

Despite the classification of vascular anomalies established by the International Society for the Study of Vascular Anomalies,^1^ the literature is not always in agreement with these different entities. Because of this confusion, and as most of the haemangiomas are asymptomatic, the incidence of CH remains unknown.

### Clinical aspects

RICH and NICH present similar clinical features except a different evolution.^3^ CH can be asymptomatic, but can also rarely lead to death as in our case.^2,3^ Aanemia, thrombocytopenia and consumptive coagulopathy are due to intralesional thrombosis.^1,2,6^ High-output cardiac failure is due to one or more large arteriovenous or portovenous shunts.^2,6^ These shunts are responsible for blood steal by the hypervascularized mass and generally close with involution.^6^ Liver failure is an uncommon complication of CHH.^2^

### Imaging

The aim of imaging is to evaluate the extension and the supplying and draining vessels with ultrasound and MRI.^2^ In case of multiple cutaneous haemangiomas or a single large one, an abdominal ultrasound is recommended to search for other visceral localizations.^2^

On ultrasound, RICH appears as well marginated and NICH as well marginated or ill marginated.^2–4,6^ RICH is hypoechoic or isoechoic, whereas NICH is isoechoic compared with the liver.^4,7^ CH is heterogeneous unlike IH because of intralesional visible vessels and calcifications.^2,4,7^ In RICH, these intratumoral calcifications increase with involution.^4,6^

On Doppler ultrasound, CH appears as a vascular mass, with high vessel density similar to IH.^3,4^ CH presents an arterial and venous vascularization, mostly veins unlike IH.^4,5^ Only internal venous vessels persist after regression of RICH.^7^ Intravascular thrombi can be seen.^5^ Intralesional arteriovenous and portovenous shunts and various-sized vascular aneurysms can be found on ultrasound and Doppler.^2,5^ Shunts are sometimes detected in NICH but rarely in RICH.^4^ Enlarged hepatic artery or veins can be observed in CH.^2^

On CT scan, CH is isodense and less well marginated than IH.^4^ RICH is homogeneous or heterogeneous, whereas NICH is heterogeneous.^4^ CH sometimes presents fat stranding unlike IH.^4^ RICH and NICH generally enhance homogeneously.^4^

On MRI, CH appears well marginated, but less so than IH.^4^ On *T*_1_ weighted images, CH appears heterogeneous and isointense compared with the liver, but NICH can also appear similarly heterogeneous as IH and isointense.^2–4^ On *T*_2_ weighted images, CH appears heterogeneous and hyperintense compared with the liver, but NICH can also appear similarly homogeneous as IH.^2–4^ Fat stranding is more frequent in NICH than RICH and is never seen in IH.^4^ Intratumoral flow voids due to high-flow vessels within and near the mass are larger in RICH than in IH and rare in NICH.^2–4^ RICH is not associated with prominent drainage veins unlike NICH.^4^ CH enhances homogeneously, similar to IH, but is not associated with peripheral oedema as in IH.^3,4^

### Differential diagnosis

RICH, NICH and IH have overlapping clinical and pathological features, especially as IH can rarely be present at birth.^3^ Larger flow voids and inhomogeneous areas help in differentiating some cases of RICH from IH.^3^ Other differential diagnoses of focal hepatic tumour in this age range includes mesenchymal hamartoma, hepatoblastoma and, in case of multiple hepatic lesions, metastasis of neuroblastoma (Pepper’s syndrome).^2^ Dilated hepatic vessels or cardiac disorders help in orientating to hepatic haemangioma.^2^

### Treatment

Only life-threatening, function-threatening or non-aesthetic haemangiomas are treated.^3,7,8^ There is actually no consensus but, generally, medical treatment (corticotherapy, propranolol, vincristine and interferon) is tried before using embolization or surgical resection.^2,6,8^

### Prognosis

During foetal life, the presence of cardiomegaly or cardiac failure, a large CH or two or three enlarged hepatic veins are bad prognostic factors.^2^ After birth, prognosis depends on complications of CH.

## Learning points

1. CHH is an uncommon neonatal benign vascular tumour.
2. Three subtypes of CH are actually described: RICH, NICH and PICH.
3. Most CHs are asymptomatic, but some CHHs can lead to death.
4. CH and IH have overlapped clinical and imaging findings.
5. The aim of imaging is to evaluate the extension and vascularization of the haemangioma.
6. Differential diagnoses of CHHs include IH, mesenchymal hamartoma, hepatoblastoma and Pepper’s syndrome in case of multiple hepatic lesions.
7. Dilated hepatic vessels or cardiac disorders help in orientating towards hepatic haemangioma.
8. There is actually no consensual treatment.

## Consent

Written informed consent for this case to be published (including images, case history and data) was obtained.
